# Breastfeeding and Bone Mass at the Ages of 18 and 30: Prospective Analysis of Live Births from the Pelotas (Brazil) 1982 and 1993 Cohorts

**DOI:** 10.1371/journal.pone.0122759

**Published:** 2015-04-16

**Authors:** Ludmila Correa Muniz, Ana Maria Baptista Menezes, Maria Cecília Formoso Assunção, Fernando Cesar Wehrmeister, Jeovany Martínez-Mesa, Helen Gonçalves, Marlos Rodrigues Domingues, Denise Petrucci Gigante, Bernardo Lessa Horta, Fernando C. Barros

**Affiliations:** 1 Post-Graduate Program in Epidemiology, Federal University of Pelotas, Pelotas, Brazil; 2 Post-Graduate Program in Physical Education, Federal University of Pelotas, Pelotas, Brazil; Institute of Preventive Medicine, DENMARK

## Abstract

**Objective:**

To evaluate the effect of total breastfeeding, breastfeeding duration and type of breastfeeding at 3 months of age on bone mass at 18 and 30 years.

**Study Design:**

A prospective, longitudinal study was conducted with two birth cohorts (1982 and 1993) in Pelotas, Southern Brazil. Measurements of bone mineral content (BMC) and bone mineral density (BMD) at 18 and 30 years of age were obtained by dual-energy X-ray absorptiometry (DXA). Information on breastfeeding was collected during the first 4 years of life. Analyses were performed by linear regression and stratified by sex.

**Results:**

A total of 1109 and 3226 participants provided complete information on breastfeeding in early life and bone mass at 18 and 30 years, respectively. No association between breastfeeding and bone mass was observed in women at both ages nor among men at age 30. Among men at the age of 18, BMC and BMD were higher among those breastfed regardless of duration (p=0.032 and p=0.043, respectively).

**Conclusions:**

Despite a very weak positive effect of breastfeeding (yes/no) on BMC and BMD at age 18 in men, most findings pointed to a lack of association between breastfeeding and bone mass until young adulthood.

## Introduction

Osteoporosis is a leading cause of morbidity and mortality worldwide, particularly because of its association with bone fractures later in life [1–3]. Bone density, a major predictor of fractures, is a result of the balance between the amount of bone mass gained earlier in life (peak bone mass) and subsequent bone loss [4].

Many genetic and environmental factors have been suggested as determinants of peak bone mass, which is attained between late adolescence and early adulthood [5, 6]. During the life-course, early eating patterns such as breastfeeding, may also influence the accumulation and subsequent preservation of bone mass [7–9]. The plausibility for a protective effect of breast milk has been described as a consequence of increased bioavailability and absorption of nutrients such as calcium and phosphorus, when compared to other types of milk [10]. Another suggested mechanism is the potentiation effect of human milk on bone development, which may be due to the presence of some non-nutritional components, such as growth factors and hormones [11]. Furthermore, early exposure to breast milk, even if for short periods, could lead to changes in the programming of bone cells, resulting in increased bone mass later in life [12].

Despite these benefits, the literature has presented conflicting results regarding the short- and long-term effects of breastfeeding on bone mineral content (BMC) and/or bone mineral density (BMD). Some studies have shown a positive effect of breastfeeding on bone mass in childhood and adolescence [10, 13–15], while others report lack of association or negative effects [16–20].

Therefore, the aim of the present study was to evaluate the effect of total breastfeeding, breastfeeding duration and type of breastfeeding at three months of age on BMC and BMD at 18 and 30 years, among individuals enrolled in the 1982 and 1993 Pelotas birth cohorts.

## Methods

### Participants

All live births from 1982 and 1993 of residents in the city of Pelotas, Rio Grande do Sul, Brazil, were considered eligible for two birth cohort studies, which included 5914 and 5249 participants, respectively. Methodological details of the studies can be found elsewhere [21–23].

**Fig 1:**
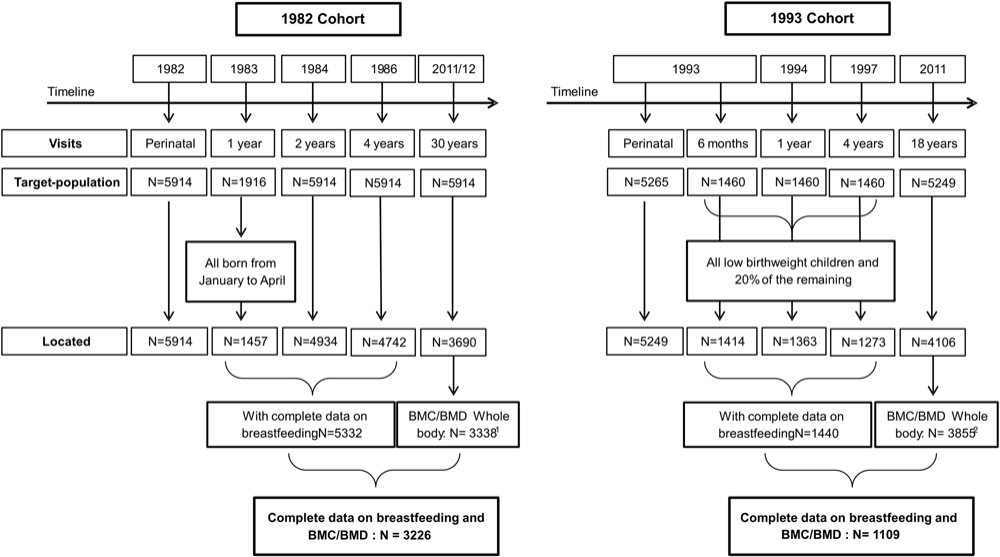
Description of the 1982 and 1993 Pelotas Birth Cohorts. 1 Subjects excluded of DXA scan: pregnant or suspected pregnancy (n = 72); individuals in wheelchairs or with osteo-articular disabilities (n = 5); losses/refusals or subjects with wearing non-removable metallic objects (e.g. screws, earrings or piercings) or extremely obese individuals or those taller than 192 cm (n = 275); 2 Subjects excluded of DXA scan: pregnant or suspected pregnancy (n = 60); individuals in wheelchairs or with osteo-articular disabilities (n = 11); losses (n = 10); subjects with wearing non-removable metallic objects (e.g. screws, earrings or piercings) or extremely obese individuals or those taller than 192 cm (n = 105).

In the present analysis, all participants of the 1982 birth cohort and young adults from a subsample of the 1993 cohort who had complete data on breastfeeding in the first four years of life were included. The subsample from the 1993 cohort included all low birth weight children (<2500g), plus a random sample of 20% of the remaining original cohort [23, 24] (Fig 1).

All follow-ups of the Pelotas Birth Cohorts were granted approval from the local Ethics Committee. At all stages, the participants (or their legal guardians) signed an informed consent form.

### Breastfeeding assessment

Breastfeeding information was obtained prospectively during the first four years of life, by home interviews with mothers. In our analysis, breastfeeding was included as a dichotomous variable (yes/no); and as an ordinal variable, in which the total duration of breastfeeding, whether exclusive, predominant or partial, was categorized in months (never; 0.01–1.00; 1.01 to 3.00; 3.01–6.00; 6.01 to 12.00; and > 12.00). The pattern of breastfeeding at three months of age was classified into four groups: "exclusive" (children who received only breast milk and no other liquid or solid foods); "Predominant" (children who received liquids other than breast milk, such as water and other water-based liquids such as fruit juice or tea); "Partial" (children who received, in addition to breast milk, food supplements with other types of milk, such as cow's milk or formulas, or semi-solid / solid food) and "weaned" (children who did not receive breast milk) [25]. For the analysis, and due to the small number of observations, we chose to combine the categories "exclusive" and "predominant" (few children were exclusively breastfed at three months of age).

### Bone mineral content and density

Our outcomes, whole body BMC (g) and BMD (g/cm^2^) were assessed at the time of follow-ups at ages 18 and 30, in the 1993 and 1982 birth cohorts, respectively. Both measures were obtained by Dual-energy X-ray Absorptiometry (DXA) (Lunar Prodigy Advance—GE, Germany) [26] and analyzed continuously. Participants presenting any of the following exclusion criteria were not examined: pregnancy or suspected pregnancy; individuals in wheelchairs; with osteo-articular disabilities; wearing non-removable metallic objects (e.g. screws, earrings or piercings); and extremely obese individuals (weight exceeding 120 kg) or those taller than 192 cm (Fig 1).

### Covariates

The following variables from the perinatal studies were used as potential confounders: family income (minimum wages); maternal education (years of schooling); maternal age (years); maternal skin color; maternal smoking during pregnancy; parity (number of previous children); gestational age at birth (weeks); birth weight (grams); and skin color. Further information about z-scores of height-for-age at 12 months (cohort 1993) and 24 months (cohort 1982) were used, according to World Health Organization for children under five [27]. Analysis on both cohorts were also adjusted for total physical activity (minutes/week); the 1993 cohort was additionally adjusted for calcium intake (mg/day adjusted for total calories) at 18 years; this latter variable was not available for the 1982 cohort. All analyses were adjusted for height and weight at the age of 18 (1993 cohort) and 30 (1982 cohort), because body size is highly correlated with bone mass.

### Statistical analyses

Statistics were performed using the *Stata* 12.1 (Stata Corp., College Station, Texas, EUA) software package and stratified by sex, given the evidence suggesting gender differences in bone mass accumulation [28, 29]. Descriptive analyses were performed using absolute and relative frequencies for categorical variables and mean and standard deviations (SD) for numeric variables (median and interquartile range for asymmetric variables). Mean and SD of outcomes according to main exposures were obtained by analysis of variance (ANOVA) or the Kruskal-Wallis test. In unadjusted and adjusted analysis, the β coefficients, 95% confidence intervals (95% CI) and p-values of the Wald’s test for heterogeneity were obtained by linear regression. When adjusting for potential confounders, the variables were included in the regression following a complete fit model, regardless of their significance level with the outcome of the bivariate analysis. Results related to the 1993 birth cohort were weighted to match the proportion of low birth weight in the overall population from the perinatal study.

## Results

Out of the 5332 participants in the 1982 cohort and 1440 young adults in the subsample of the 1993 cohort, for whom information on maternal breastfeeding in the first four years of life was available, 3226 and 1109 had undergone full body scans at 30 and 18 years of age, respectively, and were included in the analyses. Table 1 shows that, among those born in 1982, there was no statistically significant difference in most variables between participants included in the analyses and individuals not assessed (N = 2106). However, a greater proportion of individuals with a family income of less than or equal to three minimum wages (69.9% versus 68.0%), a lower percentage of male participants (47.9% versus 56.5%) and of premature newborns (5.5% versus 5.6%) and lower mean weight (74.4±15.7 kg versus 96.7±29.1 kg) and height at 30 years of age (1.67±0.09 m versus 1.71±0.11 m) was observed in individuals assessed than in those not included in the analyses. Among the participants in the 1993 cohort, a lower mean weight (65.3±13.9 kg versus 81.5±28.5 kg) was seen in participants included in the analyses than in individuals not assessed (N = 331). Moreover, the young adults were more physically active (671.9±754.7 min/week versus 470.0±567.1 min/week).

**Table 1:** Characteristics of the participants with complete data compared with participants not included in the analysis. The 1982 and 1993 Pelotas Birth Cohorts. Brazil.

	1982 (N = 5332)			1993 (N = 1440)		
	N			N		
	Mean (SD); %			Mean (SD); %		
Variables	Participants included in the analysis	Participants excluded from the analysis^1^	*p-value*	Participants included in the analysis	Participants excluded from the analysis^1^	*p-value*
	N = 3226	N = 2016		N = 1109	N = 331	
Sex	N = 3226	N = 2106	<0.001^3^	N = 1109	N = 331	0.839^3^
Males	47.9	56.5		49.8	50.5	
Females	52.1	43.5		50.2	49.5	
Family income (minimum wages)	N = 3211	N = 2098	0.010^3^	N = 1094	N = 322	0.541^3^
≤ 1	20.1	21.6		17.8	21.2	
1.1–3.0	49.8	46.4		44.3	43.1	
3.1–6.0	19.2	18.6		22.6	19.8	
>6.0	10.9	13.4		15.3	15.9	
Maternal skin color	N = 3225	N = 2104	0.772^3^	N = 1109	N = 331	0.168^3^
White	82.1	81.8		77.8	81.6	
Non-white	17.9	18.2		22.2	18.4	
Maternal education (years)	N = 3222	N = 2105	0.306^3^	N = 1106	N = 331	0.454^3^
0–4	32.4	32.5		26.3	28.8	
5–8	43.0	40.9		48.1	43.6	
9–11	10.9	11.6		17.6	17.4	
≥12	13.7	15.0		8.0	10.2	
Maternal age (years)	N = 3226	N = 2105	0.378^3^	N = 1109	N = 331	0.198^3^
< 20	14.8	16.2		14.4	18.6	
20–34	74.8	73.9		74.8	69.9	
> 34	10.4	9.9		10.8	11.5	
Maternal smoking during pregnancy	N = 3226	N = 2106	0.110^3^	N = 1109	N = 331	0.354^3^
No	65.5	63.4		67.6	64.7	
Yes	34.5	36.6		32.4	35.3	
Parity (number of previous children)	N = 3226	N = 2105	0.049^3^	N = 1109	N = 331	0.151^3^
None	39.6	38.8		33.2	37.2	
1	29.2	26.9		28.5	24.7	
2	16.1	16.6		21.3	17.1	
3	6.3	8.1		6.8	7.2	
≥4	8.8	9.6		10.2	13.8	
Gestational age at birth (weeks)	N = 2597	N = 1665	0.017^3^	N = 1104	N = 328	0.723^3^
< 34	0.5	0.6		1.7	2.6	
34–36	5.0	5.0		8.2	8.9	
37–40	68.0	72.1		88.0	86.5	
> 40	26.5	22.3		2.1	2.0	
Birth weight (grams)	N = 3225	N = 2105	0.293^3^	N = 1107	N = 330	0.420^3^
< 2500	7.1	8.3		9.9	8.1	
2500–2999	23.8	23.2		23.2	26.7	
3000–3499	38.1	38.8		41.2	40.3	
3500–3999	25.2	23.4		20.1	17.7	
≥ 4000	5.8	6.3		5.6	7.2	
Skin color	N = 3225	N = 357	0.190^3^	N = 1063	N = 165	0.477^2^
White	75.9	79.0		63.7	60.6	
Non-white	24.1	21.0		36.3	39.4	
Height-for-age at 12 months (z-score)	-	-		N = 1062	N = 297	0.812^2^
				-0.23 (1.25)	-0.28 (1.40)	
Height-for-age at 24 months (z-score)	N = 3055	N = 1876	0.499^2^	-	-	
	-0.70 (1.19)	-0.69 (1.33)				
Height at 18 years (m)	-	-		N = 1106	N = 37	0.304^2^
				1.67 (0.10)	1.71 (0.14)	
Height at 30 years (m)	N = 3222	N = 273	<0.001^2^	-	-	
	1.67 (0.09)	1.71 (0.11)				
Weight at 18 years (kg)	-	-		N = 1106	N = 37	0.014^2^
				65.34 (13.89)	81.50 (28.54)	
Weight at 30 years (kg)	N = 3222	N = 217	<0.001^2^	-	-	
	74.44 (15.72)	96.71 (29.10)				
Total physical activity at 18 years (minutes/week)	-	-		N = 1105	N = 70	0.004^2^
				671.91 (754.68)	470.04 (567.07)	
Total physical activity at 30 years (minutes/week)	N = 3178	N = 311	0.099^4^	-	-	
	300.49 (374.14)	263.67 (398.42)				
Calcium intake^5^ at 18 years (mg/day)	-	-		N = 1075	N = 62	0.255^4^
				704.95 (350.14)	755.49 (362.11)	

N: Number of observations; SD: standard deviation;

^1^Participants excluded from the analysis due to loss of follow-up or missing data;

^2^Kruskal-Wallis;

^3^Chi-square test;

^4^Analysis of variance;

^5^No data for 1982 cohort study

Among those born in 1982 and 1993, females accounted for 52.1% and 50.2% of the samples, respectively, and had lower BMC and BMD when compared to males: mean BMC and BMD at 30 years was 2544.6 ±391.6g and 1.165 ±0.08g/cm^2^ in women versus 3195.4 ±466.4g and 1.274 ±0.10g/cm^2^ in men, respectively, while mean values of BMC and BMD at 18 years were 2411.65 ±404.6g and 1.135 ±0.08g/cm^2^ in women versus 2947.9 ±468.2g and 1.222 ±0.10g/cm^2^ in men, respectively (p <0.001 for both outcomes and studies) (Table 2).

**Table 2:** Descriptive analysis of the whole body bone mineral content (BMC) and bone mineral density (BMD) at ages 18 and 30 according to breastfeeding variables, stratified by sex. The 1982 and 1993 Pelotas Birth Cohorts. Brazil.

	*Men*					
	1982			1993		
Variables	Mean (SD)			Mean (SD)		
	N	BMC (g)	BMD (g/cm^2^)	N	BMC (g)	BMD (g/cm^2^)
	1545	3195.39 (466.37)	1.274 (0.096)	531	2947.91 (468.21)	1.222 (0.097)
Breastfeeding		*p = 0*.*913* ^*1*^	*p = 0*.*703* ^*1*^		*p = 0*.*381* ^*2*^	*p = 0*.*292* ^*2*^
No	125	3199.76 (491.30)	1.277 (0.105)	35	2803.96 (366.35)	1.196 (0.070)
Yes	1420	3195.00 (464.29)	1.274 (0.095)	496	2954.00 (471.55)	1.224 (0.098)
Total duration of breastfeeding (months)		*p = 0*.*029* ^*1*^	*p = 0*.*007* ^*1*^		*p = 0*.*052* ^*2*^	*p = 0*.*027* ^*2*^
Never	125	3199.76 (491.30)	1.277 (0.105)	35	2803.96 (366.35)	1.196 (0.070)
0.01–1.00	361	3145.12 (468.44)	1.261 (0.094)	95	3090.23 (540.07)	1.247 (0.108)
1.01–3.00	460	3195.27 (473.42)	1.274 (0.098)	160	2920.20 (429.24)	1.215 (0.088)
3.01–6.00	217	3164.75 (439.90)	1.269 (0.092)	93	2889.71 (453.10)	1.215 (0.093)
6.01–12.00	162	3271.34 (450.59)	1.290 (0.088)	59	3049.96 (489.83)	1.255 (0.102)
> 12.00	220	3249.94 (462.42)	1.286 (0.096)	76	2892.31 (452.81)	1.214 (0.099)
Pattern of breastfeeding at three months of age		*p = 0*.*042* ^*1*^	*p = 0*.*023* ^*1*^		*p = 0*.*175* ^*1*^	*p = 0*.*6320* ^*1*^
Weaning	740	3164.93 (473.37)	1.267 (0.098)	245	2977.56 (479.65)	1.222 (0.097)
Partial	395	3214.21 (465.13)	1.280 (0.095)	122	2883.66 (445.34)	1.218 (0.096)
Exclusive/predominant	410	3232.23 (452.13)	1.280 (0.094)	157	2962.30 (470.32)	1.228 (0.097)
	***Women***					
	**1982**			**1993**		
	**Mean (SD)**			**Mean (SD)**		
	**N**	**BMC (g)**	**BMD (g/cm** ^**2**^ **)**	**N**	**BMC (g)**	**BMD (g/cm** ^**2**^ **)**
	1681	2544.59 (391.58)	1.165 (0.078)	576	2411.65 (404.55)	1.135 (0.082)
Breastfeeding		*p = 0*.*262* ^*1*^	*p = 0*.*291* ^*1*^		*p = 0*.*025* ^*2*^	*p = 0*.*184* ^*1*^
No	128	2507.30 (384.33)	1.158 (0.076)	26	2290.12 (331.74)	1.108 (0.099)
Yes	1553	2547.67 (392.14)	1.166 (0.078)	550	2414.60 (406.12)	1.136 (0.081)
Total duration of breastfeeding (months)		*p = 0*.*676* ^*1*^	*p = 0*.*732* ^*1*^		*p = 0*.*320* ^*1*^	*p = 0*.*219* ^*1*^
Never	128	2507.30 (384.33)	1.158 (0.076)	26	2290.12 (331.74)	1.108 (0.099)
0.01–1.00	365	2557.23 (402.01)	1.163 (0.081)	97	2391.21 (422.21)	1.122 (0.085)
1.01–3.00	519	2534.08 (384.43)	1.166 (0.075)	165	2377.51 (385.50)	1.129 (0.077)
3.01–6.00	240	2548.04 (398.01)	1.165 (0.075)	91	2433.00 (430.85)	1.140 (0.078)
6.01–12.00	178	2576.78 (378.28)	1.171 (0.077)	66	2489.02 (397.23)	1.143 (0.084)
> 12.00	251	2540.87 (398.97)	1.167 (0.086)	121	2425.02 (418.22)	1.144 (0.083)
Pattern of breastfeeding at three months of age		*p = 0*.*287* ^*1*^	*p = 0*.*446* ^*1*^		*p = 0*.*583* ^*1*^	*p = 0*.*058* ^*1*^
Weaning	784	2535.16 (389.71)	1.163 (0.077)	230	2389.62 (393.36)	1.124 (0.082)
Partial	432	2535.45 (378.99)	1.166 (0.077)	158	2411.42 (389.90)	1.136 (0.075)
Exclusive/predominant	465	2569.00 (405.78)	1.168 (0.082)	184	2431.64 (432.07)	1.144 (0.086)

SD: standard deviation;

^1^Analysis of variance;

^2^Kruskal-Wallis

With respect to the exposures of interest, among those born in 1982, around 8.0% were not breastfed (7.6% of girls and 8.1% of boys) whereas among those born in 1993, 5.0% of the sample never received breast milk (2.8% of girls and 4.4% of boys). At three months, 47.2% of births in 1982 and 40.1% of those born in 1993 had already been weaned. The median total duration of breastfeeding was 3.0 months [25^th^ Percentile (P25): 1.0 – 75^th^ Percentile (P75): 6.0] among boys and 3.0 months (P25: 1.0—P75: 7.0) among girls born in 1982 compared with 3.0 months (P25: 1.5—P75: 8.0) among boys and 3.5 months (P25: 1.5—P75 12.0) among girls born in 1993.

Table 2 presents the descriptive analysis of outcomes according to breastfeeding variables, stratified by sex, for both studies. Among men, the variables full breastfeeding in months and pattern of breastfeeding at three months, were associated with both outcomes at 30 years of age. When bone mass was assessed at 18 years, only total breastfeeding (in months) was associated with BMD (p = 0.027), but with no dose-response effect. Among women, no breastfeeding variables were associated with outcomes assessed at 30 years of age; however at the age of 18, women who had been breastfed, regardless of duration, had higher BMC compared to those who were never breastfed (p = 0.025).

Table 3 displays the crude and adjusted coefficients of the associations between breastfeeding and BMC at 18 and 30 years for men and women. No evidence was observed between breastfeeding variables and BMC in women in both studies. Among men, higher values of BMC were observed at age 18 among those who had been breastfed for any period of time compared to those never breastfed (crude and adjusted analyses). Also among males, the total maternal breastfeeding variable in months was associated, in the crude analysis only, with BMC at 18 and 30 years, although the association did not exhibit a tendency pattern. Higher BMC at 30 years was also observed among men exclusively/predominantly fed maternal milk up to the third month of life, compared to those who had already been weaned. However, this effect disappeared after adjusting for confounding factors.

**Table 3:** Crude and adjusted coefficients of the association between breastfeeding variables and whole body bone mineral content (BMC) at 18 and 30 years for men and women. The 1982 and 1993 Pelotas Birth Cohorts. Brazil.

	*Men*			
	1982		1993	
Variables	(β; 95%CI)		(β; 95%CI)	
	Crude	Adjusted^1^	Crude	Adjusted^2^
Breastfeeding	*p = 0*.*913*	*p = 0*.*856*	*p = 0*.*036*	*p = 0*.*032*
No	Ref.	Ref.	Ref.	Ref.
Yes	-4.75 (-90.13; 80.62)	-6.14 (-72.49; 60.21)	150.94 (9.78; 292.11)	125.51 (11.09; 239.92)
Total duration of breastfeeding (months)	*p = 0*.*029*	*p = 0*.*389*	*p = 0*.*014*	*p = 0*.*120*
Never	Ref.	Ref.	Ref.	Ref.
0.01–1.00	-54.64 (-149.35; 40.07)	-24.08 (-98.22; 50.07)	287.77 (105.03; 470.50)	146.75 (17.61; 275.90)
1.01–3.00	-4.49 (-96.54; 87.56)	-7.42 (-78.84; 63.99)	117.05 (-34.60; 268.71)	105.82 (-11.64; 223.29)
3.01–6.00	-35.00 (-137.47; 67.47)	-18.94 (-97.46; 59.58)	87.06 (-78.39; 252.51)	138.66 (10.92; 266.41)
6.01–12.00	71.58 (-37.06; 180.23)	-13.37 (-96.96; 70.22)	246.83 (62.03; 431.63)	169.99 (32.72; 307.27)
> 12.00	50.19 (-52.03; 152.40)	42.64 (-36.87; 122.15)	88.90 (-83.09; 260.88)	73.26 (-60.43; 206.94)
Pattern of breastfeeding at three months of age	*p = 0*.*042*	*p = 0*.*304*	*p = 0*.*205*	*p = 0*.*824*
Weaning	Ref.	Ref.	Ref.	Ref.
Partial	49.28 (-7.64; 106.20)	29.91 (-13.25; 73.06)	-94.12 (-201.27; 13.03)	16.78 (-47.70; 81.26)
Exclusive/predominant	67.30 (11.06; 123.54)	26.83 (-17.05; 70.70)	-15.20 (-117.32; 86.92)	17.35 (-48.25; 82.95)
	***Women***			
	**1982**		**1993**	
	**(β; 95%CI)**		**(β; 95%CI)**	
	**Crude**	**Adjusted** ^1^	**Crude**	**Adjusted** ^2^
Breastfeeding	*p = 0*.*262*	*p = 0*.*289*	*p = 0*.*111*	*p = 0*.*366*
No	Ref.	Ref.	Ref.	Ref.
Yes	40.37 (-30.25; 110.99)	29.99 (-25.44; 85.42)	122.55 (-28.22; 273.32)	54.42 (-63.70; 172.55)
Total duration of breastfeeding (months)	*p = 0*.*676*	*p = 0*.*199*	*p = 0*.*282*	*p = 0*.*679*
Never	Ref.	Ref.	Ref.	Ref.
0.01–1.00	49.93 (-29.01; 128.87)	60.79 (-0.85; 122.43)	99.20 (-75.00; 273.40)	46.85 (-77.97; 171.67)
1.01–3.00	26.78 (-49.06; 102.62)	20.51 (-38.57; 79.59)	85.27 (-74.54; 245.08)	43.73 (-76.99; 164.45)
3.01–6.00	40.74 (-43.37; 124.85)	10.55 (-54.72; 75.81)	141.51 (-35.33; 318.34)	68.47 (-61.41; 198.35)
6.01–12.00	69.48 (-19.58; 158.54)	40.77 (-28.53; 110.07)	197.05 (19.54; 374.56)	29.52 (-102.17; 161.21)
> 12.00	33.57 (-49.89; 117.04)	15.68 (-49.29; 80.65)	132.74 (-33.70; 299.17)	74.82 (-49.44; 199.09)
Pattern of breastfeeding at three months of age	*p = 0*.*287*	*p = 0*.*635*	*p = 0*.*648*	*p = 0*.*606*
Weaning	Ref.	Ref.	Ref.	Ref.
Partial	0.29 (-45.73; 46.30)	-16.60 (-51.55; 18.35)	21.22 (-65.79; 108.23)	13.87 (-36.38; 64.11)
Exclusive/predominant	33.84 (-11.11; 78.79)	-9.88 (-44.87; 25.11)	42.11 (-47.15; 131.37)	24.31 (-23.72; 72.34)

β, regression coefficient. 95%CI, 95% confidence interval; *p-value*: *Wald’s test for heterogeneity*

^1^Adjusted for maternal education, maternal age, family income, maternal smoking during pregnancy, gestational age, birth weight, z-score of height-for-age at 24 months, maternal skin color, parity and total physical activity, weight and height at 30 years;

^2^Adjusted for maternal education, maternal age, family income, maternal smoking during pregnancy, gestational age, birth weight, z-score of height-for-age at 12 months, maternal skin color, parity and total physical activity, calcium intake, weight and height at 18 years

Table 4 presents the crude and adjusted coefficients of the association between breastfeeding variables and BMD at 18 and 30 years, stratified by sex. In females, only categorical breastfeeding in months showed an association with BMD at the age of 18. However, the effect was lower and statistical significance was no longer observed after adjusting for potential confounders. In men, as observed for BMC, only crude analysis resulted in statistically significant associations between categorical breastfeeding and BMD at 30 years. Among men aged 18 years, using dichotomous breastfeeding, both crude and adjusted analyses showed that BMD was higher among those who had been breastfed. When analyzing total breastfeeding (in months), similar results for BMC were observed. A higher BMD at 18 years was found among men breastfed for less than one month (β crude = 0.051 95%CI 0.016; 0.087) and from 6.01 to 12 months (β crude = 0.060 95%CI 0.023; 0.097) compared to those not breastfed, where this effect disappeared after adjusting for confounders.

**Table 4:** Crude and adjusted coefficients of the association between breastfeeding variables and whole body bone mineral density (BMD) at 18 and 30 years for men and women. The 1982 and 1993 Pelotas Birth Cohorts. Brazil.

	*Men*			
	1982		1993	
Variables	(β; 95%CI)		(β; 95%CI)	
	Crude	Adjusted^1^	Crude	Adjusted^2^
Breastfeeding	*p = 0*.*703*	*p = 0*.*734*	*p = 0*.*042*	*p = 0*.*043*
No	Ref.	Ref.	Ref.	Ref.
Yes	-0.003 (-0.021; 0.014)	-0.003 (-0.022; 0.015)	0.028 (0.001; 0.056)	0.026 (0.001; 0.050)
Total duration of breastfeeding (months)	*p = 0*.*007*	*p = 0*.*205*	*p = 0*.*009*	*p = 0*.*116*
Never	Ref.	Ref.	Ref.	Ref.
0.01–1.00	-0.016 (-0.036; 0.003)	-0.011 (-0.032; 0.009)	0.051 (0.016; 0.087)	0.030 (-0.001; 0.060)
1.01–3.00	-0.003 (-0.022; 0.016)	-0.003 (-0.023; 0.016)	0.019 (-0.010; 0.049)	0.018 (-0.008; 0.044)
3.01–6.00	-0.008 (-0.029; 0.013)	-0.007 (-0.029; 0.015)	0.019 (-0.013; 0.052)	0.028 (-0.001; 0.057)
6.01–12.00	0.013 (-0.010; 0.035)	-0.001 (-0.022; 0.024)	0.060 (0.023; 0.097)	0.044 (0.011; 0.076)
> 12.00	0.009 (-0.012; 0.030)	0.011 (-0.011; 0.033)	0.018 (-0.017; 0.053)	0.015 (-0.017; 0.047)
Pattern of breastfeeding at three months of age	*p = 0*.*023*	*p = 0*.*253*	*p = 0*.*676*	*p = 0*.*479*
Weaning	Ref.	Ref.	Ref.	Ref.
Partial	0.013 (0.001; 0.025)	0.009 (-0.003; 0.021)	-0.005 (-0.027; 0.018)	0.001 (-0.013; 0.026)
Exclusive/predominant	0.014 (0.002; 0.025)	0.008 (-0.004; 0.020)	0.006 (-0.015; 0.027)	0.011 (-0.007; 0.030)
	***Women***			
	**1982**		**1993**	
	**(β; 95%CI)**		**(β; 95%CI)**	
	**Crude**	**Adjusted** ^1^	**Crude**	**Adjusted** ^2^
Breastfeeding	*p = 0*.*291*	*p = 0*.*576*	*p = 0*.*285*	*p = 0*.*472*
No	Ref.	Ref.	Ref.	Ref.
Yes	0.008 (-0.007; 0.022)	0.004 (-0.010; 0.018)	0.027 (-0.023; 0.077)	0.016 (-0.027; 0.059)
Total duration of breastfeeding (months)	*p = 0*.*732*	*p = 0*.*910*	*p = 0*.*028*	*p = 0*.*408*
Never	Ref.	Ref.	Ref.	Ref.
0.01–1.00	0.005 (-0.011; 0.020)	0.006 (-0.010; 0.021)	0.014 (-0.039; 0.067)	0.010 (-0.035; 0.055)
1.01–3.00	0.007 (-0.008; 0.023)	0.004 (-0.011; 0.019)	0.021 (-0.030; 0.072)	0.012 (-0.032; 0.056)
3.01–6.00	0.006 (-0.010; 0.023)	-0.001 (-0.017; 0.016)	0.031 (-0.022; 0.084)	0.021 (-0.024; 0.066)
6.01–12.00	0.013 (-0.005; 0.031)	0.007 (-0.011; 0.024)	0.034 (-0.020; 0.088)	0.010 (-0.039; 0.056)
> 12.00	0.009 (-0.007; 0.026)	0.004 (-0.012; 0.021)	0.035 (-0.017; 0.087)	0.025 (-0.020; 0.070)
Pattern of breastfeeding at three months of age	*p = 0*.*446*	*p = 0*.*946*	*p = 0*.*105*	*p = 0*.*268*
Weaning	Ref.	Ref.	Ref.	Ref.
Partial	0.004 (-0.005; 0.013)	-0.001 (-0.009; 0.008)	0.012 (-0.006; 0.029)	0.010 (-0.007; 0.024)
Exclusive/predominant	0.006 (-0.003; 0.015)	-0.002 (-0.010; 0.007)	0.019 (0.001; 0.037)	0.012 (-0.003; 0.026)

β, regression coefficient. 95%CI, 95% confidence interval; *p-value*: *Wald’s test for heterogeneity*

^1^Adjusted for maternal education, maternal age, family income, maternal smoking during pregnancy, gestational age, birth weight, z-score of height-for-age at 24 months, maternal skin color, parity and total physical activity, weight and height at 30 years;

^2^Adjusted for maternal education, maternal age, family income, maternal smoking during pregnancy, gestational age, birth weight, z-score of height-for-age at 12 months, maternal skin color, parity and total physical activity, calcium intake, weight and height at 18 years

## Discussion

Current evidence shows that the effect of breastfeeding on bone mass may extend beyond childhood, in accordance with Barker’s hypotheses [13, 20]. With respect to the main results of our study, after adjusting for potential confounders, we failed to detect any effect from the exposure to breast milk on bone health of women in both the cohorts or in men at 30 years of age. In males, only the maternal breastfeeding variable (yes/no) was associated with bone mass at 18 years of age, with greater BMC and BMD at 18 years observed among those who had been breastfed for any length of time compared to those never breastfed.

However, exposure to breast milk at least once in a lifetime was enough to classify the individual as breastfed, which is a weakness of this variable; thus, the positive effect observed in males should be interpreted with caution.

Although the effects of early dietary exposures on body composition in later life are well described in the literature, the relationship between breastfeeding and bone mineral mass is still poorly understood. Only two studies indicated a positive effect of breastfeeding on bone health in late adolescence [13, 15]. Jones et al., [13] evaluated 415 adolescents (16 years) from a Tasmanian cohort. The study showed that infants who were born full-term and breastfed had higher BMD (whole body, lumbar spine lumbar and femoral neck) compared to non-breastfed infants, and larger effects were observed among those breastfed for longer (above three months). In the study by Molgaard et al., [15] the authors also observed a direct relationship between the duration of breastfeeding and bone mass at 17 years of age. Both studies indicated a dose-response relationship between breastfeeding length and bone mass, in contrast with that observed in the present study. Several hypotheses have been postulated to explain the beneficial effect of breastfeeding and prolonged breastfeeding. The most common theory implicates greater bioavailability and absorption of calcium and phosphorus in human milk compared to other types of milk [10]. Moreover, the consumption of breast milk results in an average intake of 200mg of calcium per day [30, 31], sufficient to promote good skeletal development in childhood, which can be observed later in life [32]. Also, the presence of hormones and growth factors in human milk may enhance its effect on bone development [11] while early and prolonged exposure to breast milk may lead to changes in the programming of bone cells, resulting in long-term increased bone mass [12].

As previously mentioned, the studies that found an association between breastfeeding and bone mass [13, 15] reported positive effects of breast milk in both men and women. In the present study, no effect of breastfeeding was observed on BMC and BMD in women at 18 and 30 years of age. In this regard, some authors have suggested that among women, unlike men, hormonal factors, lifestyle, such as smoking, physical activity, calcium intake and body composition are more important for bone health than early dietary characteristics such as breastfeeding [33]. In an attempt to test this hypothesis, adjustments to mediators such as physical activity level, daily calcium intake, and body mass index at 18 and 30 years of age were made. However, after these adjustments results were unchanged. Other analyses were also performed to elucidate the lack of association among women. Initially, we assumed that as men are taller than women (about 1.61m and 1.74 m, respectively, in both studies, p <0.001; data not shown) and given that bone mass correlates with height, this could be an explanation for the observed difference between sexes regarding breastfeeding and bone mass. However, the height of the participants did not differ according to breastfeeding variables and was not identified as a mediator of the relationship between breastfeeding and bone mass. In conclusion, we could not fully explain the differences between men and women and cannot rule out the possibility that the positive results observed in men at the age of 18 were due to chance.

A limitation of this study was that exclusive breastfeeding was not analyzed dichotomously. However, we chose not to use the dichotomous variable because, at the time of the studies, the policy on breastfeeding in Brazil was nonexistent or relatively recent [34] and the median length of exclusive breastfeeding among children from the 1982 and 1993 Pelotas birth cohorts was less than a week [35]. Another reason for not using the dichotomous variable was owing to the small percentage of participants in the 1993 cohort exclusively breastfed at three months of age (only 7.0%), a much lower proportion than for those born in 1982. Consequently, a cut-off point of three months was adopted for the pattern of breastfeeding variable.

Another limitation of the present study was the lack of adjustment for daily calcium intake at 30 years in the 1982 cohort, because this information was unavailable. This latter factor may represent a significant limitation to elucidating the relationship between breastfeeding and bone mass at 30 years of age, given that calcium is a key nutrient for the formation and subsequent maintenance of bones.

Furthermore, the fact that only one subsample of the 1993 cohort was analyzed may also be regarded as a limitation of the study, in as far as a smaller sampling size may have impacted the power of the study; however, the results obtained from the subsample proved similar to those observed for the 1982 cohort which included all the participants.

On the other hand, the positive aspects of this study hinge on the prospective nature of data collection, for both exposures and confounders, thus ruling out recall bias. Another strength of this study was that measures of BMC and BMD were obtained via DXA, considered the gold standard by the International Society for Clinical Densitometry [36] to assess bone mass. Another important characteristic was the age at which the outcome was measured, as most studies published to date have evaluated bone mass during childhood [10, 16, 17]. If bone mass is measured in late adolescence, a period when many intense and rapid body changes occur impacting body composition in adulthood [37], then this allows the assessment of whether the effects of early nutrition extend beyond childhood.

## Conclusions

The present study investigated the effect of breastfeeding on BMC and BMD assessed by DXA, a technique considered the gold standard for assessing bone mass in two birth cohorts.

Although a very weak positive association was observed for the variable breastfeeding (yes or no) among men at 18 years old, these results were not consistent between the two periods; most of the findings from this study failed to show a direct association between breastfeeding and long-term effects on bone mass.
